# Enhanced production of select phytocannabinoids in medical *Cannabis* cultivars using microbial consortia

**DOI:** 10.3389/fpls.2023.1219836

**Published:** 2023-08-31

**Authors:** Bulbul Ahmed, František Beneš, Jana Hajšlová, Lenka Fišarová, Miroslav Vosátka, Mohamed Hijri

**Affiliations:** ^1^African Genome Center, Mohammed VI Polytechnic University (UM6P), Ben Guerir, Morocco; ^2^Institut de Recherche en Biologie Végétale, Université de Montréal, Montréal, QC, Canada; ^3^Department of Food Analysis and Nutrition, University of Chemistry and Technology, Prague, Czechia; ^4^Institute of Botany, Czech Academy of Sciences, Průhonice, Czechia

**Keywords:** *Cannabis*, arbuscular mycorrhizal fungi, microbiome, phytocannabinoids, rhizosphere, bioinoculant

## Abstract

The root microbiome of medical cannabis plants has been largely unexplored due to past legal restrictions in many countries. Microbes that live on and within the tissue of *Cannabis sativa* L. similar to other plants, provide advantages such as stimulating plant growth, helping it absorb minerals, providing protection against pathogen attacks, and influencing the production of secondary metabolites. To gain insight into the microbial communities of *C. sativa* cultivars with different tetrahydrocannabinol (THC) and cannabidiol (CBD) profiles, a greenhouse trial was carried out with and without inoculants added to the growth substrate. Illumina MiSeq metabarcoding was used to analyze the root and rhizosphere microbiomes of the five cultivars. Plant biomass production showed higher levels in three of five cultivars inoculated with the arbuscular mycorrhizal fungus *Rhizophagus irregularis and* microbial suspension. The blossom dry weight of the cultivar THE was greater when inoculated with *R. irregularis* and microbial suspension than with no inoculation. Increasing plant biomass and blossom dry weight are two important parameters for producing cannabis for medical applications. In mature *Cannabis*, 12 phytocannabinoid compounds varied among cultivars and were affected by inoculants. Significant differences (*p* ≤ 0.01) in concentrations of cannabidivarinic acid (CBDVA), cannabidivarin (CBDV), cannabigerol (CBG), cannabidiol (CBD), and cannabigerolic acid (CBGA) were observed in all *Cannabis* cultivars when amended with F, K1, and K2 inoculants. We found microbes that were shared among cultivars. For example, *Terrimicrobium* sp., *Actinoplanes* sp., and *Trichoderma reesei* were shared by the cultivars ECC-EUS-THE, CCL-ECC, and EUS-THE, respectively. *Actinoplanes* sp. is a known species that produces phosphatase enzymes, while *Trichoderma reesei* is a fungal train that produces cellulase and contributes to organic matter mineralization. However, the role of *Terrimicrobium* sp. as an anaerobic bacterium remains unknown. This study demonstrated that the use of inoculants had an impact on the production of phytocannabinoids in five *Cannabis* cultivars. These inoculants could have useful applications for optimizing cannabis cultivation practices and increasing the production of phytocannabinoids.

## Introduction

*Cannabis sativa* L. produces a number of valuable natural products in its fiber, grain, and flower extracts (Andre et al., 2016; Backer et al., 2019). It is thought to have been used medically for over two millennia. Through generations, genetic variability in cannabis has spread, leading to a broad range of varieties with distinct phenotypic qualities and secondary metabolites. Cannabis extracts contain metabolic components that have medical and pharmaceutical uses. The most common uses of medical cannabis include reducing chronic pain in adults caused by multiple sclerosis, post-traumatic stress disorder, cancer, epilepsy, and nausea, among others (reviewed by the National Academies of Sciences, Engineering, and Medicine, 2017). Cannabis plants contain many phytocannabinoids, which are being researched for their therapeutic properties. Two primary ones are the Δ9-tetrahydrocannabinol (THC) and cannabinol (CBN), while the others, such as cannabidiol (CBD), cannabidiol-carboxylic acid, cannabigerol (CBG), cannabichromene (CBC), are undergoing extensive investigation (Carvalho et al., 2022).

Biomass production and metabolic profiles of cannabis are influenced by growing substrates, light, temperature, fertilizer inputs (Coffman and Gentner, 1977; Winston et al., 2014; Bernstein et al., 2019; Danziger and Bernstein, 2021), and microorganisms that live on or inside plant tissues (Taghinasab and Jabaji, 2020). Cannabis hosts distinct microbial communities (neutral, beneficial, or pathogenic) on and within its tissues, designated the plant microbiota, from the moment they are planted in the soil as seeds. The plant microbiota is composed of specific microbial communities associated with the roots and the rhizosphere soil, the phyllosphere, and the internal tissues of the plant, known as the endosphere. Seeds harbor diverse groups of microbiota, which are transmitted to juvenile plants, promoting protection against biotic and abiotic stresses at seed germination and later stages (Taghinasab and Jabaji, 2020). Moreover, microbial inoculants may have the potential to increase the cannabis biomass and increase the biosynthesis of desired metabolites. This is because microorganisms release signal molecules that trigger the biosynthesis of plant biochemical compounds, including growth-stimulating substances and secondary metabolites (Ahmed and Hijri, 2021). To our knowledge, there are very limited research data regarding the impact of microbial inoculants on yield and secondary metabolite production of cannabis. A consortium of plant growth-promoting rhizobacteria (PGPR) comprising *Gluconacetobacter diazotrophicus*, *Azospirillum brasilense*, *Burkholderia ambifaria*, and *Herbaspirillum seropedicae* showed the growth improvement and accumulation of secondary metabolites in hemp (Pagnani et al., 2018). Another study evaluated the effect of a commercial microbial bioinoculant (Mammoth PTM containing beneficial bacteria) on cannabis production in soil-less systems. Hydroponically introduced Mammoth PTM increased bud yield by 16.5%; however, inoculation had not been studied for its effect on biosynthesis of phytocannabinoids (Conant et al., 2017). Therefore, we studied the impact of different microbial consortia on the yield of biomass and the biosynthesis of phytocannabinoids in five *Cannabis* cultivars.

Plant genotype influences the rhizosphere’s microbial communities because different compartments have different physical and chemical characteristics that affect the microbes in the rhizosphere, and plant roots release a wide range of chemical substances to attract and choose microbes in the rhizosphere (Berendsen et al., 2012; Marques et al., 2014; Sapkota et al., 2015). Genotypes can have a unique core microbiome (a group of microbial taxa that are always associated with a specific host). For example, soybean inoculated with a microbial suspension from forest soil increased the total phosphate accumulation by 23%, providing phytate as the sole phosphate source due to a core microbiome that was correlated with phytate mineralization (Ahmed et al., 2021a). Identifying the core microbiome of a particular genotype, which is associated with its optimal growth and nutrition, could be a diagnostic test to explain suboptimal plant performance. It might also reveal a lack of essential microorganisms that should be added to improve plant growth and enhance other desired functions, including the biosynthesis of secondary compounds (Ahmed and Hijri, 2021). Our previous study on hemp grown in six locations in New York State in agricultural production systems identified four bacterial (*Gimesia maris*, *Pirellula* sp. *Lacipirellula limnantheis*, and *Gemmata* sp.) and three fungal (*Fusarium oxysporum*, *Gibellulopsis piscis*, and *Fusarium equiseti*) core microbiome (Ahmed et al., 2021b).

Chemical profiling is essential to support botanical analysis. Cannabis varieties are typically classified based on the chemical compounds they contain, such as delta-9-tetrahydrocannabinol (THC) or cannabidiol (CBD). In recent years, CBD has become the preferred component of medicinal cannabis products due to its nonpsychotropic, anxiolytic, antispasmodic, and antiemetic pharmacological characteristics. Cannabis strains with high levels of CBD are classified as hemp (fiber and seed productions) or fiber types, whereas those with high levels of THC are classified as marijuana or narcotic types. Cannabis plants are considered intermediate when their THC/CBD ratio is approximately 1 (Carvalho et al., 2022). In this study, THC-, CBD-, and intermediate-type cultivars are examined to determine the effect of microbial bioinoculants on biomass production and associated microbial communities.

Numerous studies have tested the effects of microbial inoculants on the growth and health of cannabis plants, including industrial hemp, recreational cannabis, and medicinal cannabis. However, no study has compared different formulations of inoculants on different cannabis genotypes (Conant et al., 2017; Pagnani et al., 2018). Furthermore, the impact of introduced inoculants on indigenous microbial communities associated with cannabis remains unknown. In this study, we tested microbial inoculants on the growth and biosynthesis of 12 phytocannabinoid metabolites in five medical *Cannabis* cultivars (*Cannabis sativa* L.) grown in a greenhouse for 3 months. *C. sativa* L. is an annual, photoperiod-dependent plant that has two phases in its life cycle: vegetative growth and flowering. When given 16 or more hours of daily light, the cannabis plant undergoes vegetative growth. When the daily light hours are reduced to 12 or less, flowering begins. Because of the strong correlation between plant size and floral biomass and the timing of photoperiod switches, most commercial cannabis is grown in indoor facilities in order to better control environmental conditions (Dang et al., 2021). Therefore, this experiment was conducted in a controlled greenhouse environment. We used a coconut coir-based substrate, as it has become much more economical than other growing media such as rockwool. In addition, the coir-based substrate is easier to maintain when accounting for humidity levels and is also naturally recyclable. We hypothesized that amending medicinal cannabis plants with microbe-based bioinoculants will influence the growth and concentration of phytocannabinoids and their associated microbial community structure. To evaluate this hypothesis, we assessed the composition and structure of the microbiome in the root and rhizosphere soil by amplicon sequencing of the bacterial 16S rDNA and fungal ITS in the root and rhizosphere soils.

## Materials and methods

### Experimental design, treatments, and management

The experiment was a completely randomized design with four inoculant treatments and five cultivars of *Cannabis sativa* L.: CBD Therapy (THE), Euforia (EEA), Critical (CCL), CBD Sweet and Sour Widow (ECC), and CBD US (EUS) in a greenhouse trial of 3 months (from 7 October 2019 to 7 January 2020). Plants were grown from rooted clone cuttings in the greenhouse in 50-cell-deep flats and then transplanted to 15-L plastic pots filled with nonsterile commercial growing substrate, a mixture of coconut fiber (BioBizz Worldwide, Bizkaia, Spain) and agro perlite as a substrate (1:1). Five replicates were prepared for each inoculant × cultivar combination, for a total of 100 pots. The four inoculant treatments in this study were as follows: Ferticann (F, mixture of beneficial microbes containing 5,000 spores/g (0–120 µm, carrier silicate) of *Rhizophagus irregularis*, 10^9^ CFU/g (0–120 µm and carrier dextrose) of *Trichoderma harzianum* and *Bacillus subtilis*, and microalga *Dictyosphaerium chlorelloides*); microbial suspension from forest soil (K1) dominated by planctobacteria described by Ahmed et al. (2021a); forest microbial suspension plus *R. irregularis* isolate DAOM 197198 (K2); and control (K0) without any inoculants. The wavelength and duration of light, humidity, and temperature are all key factors for successful cannabis growth during different growth stages. We followed the cultivation conditions described by Folina et al. (2019) for growing medicinal cannabis, including the production process based on clonal plants until dried flower blossom. For each stage, plants were grown under specific light and humidity conditions. The light spectrum and intensity were adjusted to maximize flowering quality and optimize growth (Folina et al., 2019). Microbial suspensions were applied once in the soil to the base of the plants while they were being established in the growing media. To keep the substrate moist, plants were irrigated twice a week with tap water, and each pot was fertilized once a week in the morning with 50 mL of full-strength Long Ashton nutrient solution (Davies et al., 2000).

### Sample collection and plant biomass analysis

Cannabis plants were harvested after 90 days of growth. Shoots were cut at the plant collar with a clean and sterile scalpel and placed in a paper bag. Roots were separated from the growth substrate, washed with tap water, and rinsed with sterile distilled water. About 5 g of rhizospheric substrate attached to the roots was collected from each pot by gently brushing the roots in a 15-mL falcon tube for DNA extraction. Shoot and root samples were placed in paper bags, transported to the laboratory on ice, and immediately measured for shoot fresh weight, root fresh weight, and plant height. Flower blossoms were carefully separated, and shoot, root, and blossom samples were dried in a drying oven at 65°C, for 72 h, and their dry weights were also measured. Samples for DNA extraction were kept at 4°C before being brought to the laboratory and preserved at −80°C.

### Root staining to assess mycorrhizal colonization

Composite root samples from plants were rinsed under tap water to remove soil debris and cut into small pieces (~1 cm long). They were then stained and visualized under a microscope according to the protocol regularly used in our lab (Dagher et al., 2020). The roots were washed with water and cleared in 10% KOH solution. The process was repeated as necessary, the solution was changed, and the transparency of the roots was checked regularly. After 1 week, roots were washed with water, immersed in 2% lactic acid, and heated at 90°C for 20 min. After draining the lactic acid, the roots were stained with 0.05% trypan blue solution in lactoglycerol (40% glycerol and 16% lactic acid in distilled water) at 90°C for 15 min and at room temperature (20°C plusmn; 2°C) overnight. Washed roots were stored in lactoglycerol and observed in a microscope at a magnification of ×200.

### Phytocannabinoid profiling

Phytocannabinoid profiling was done on a representative sample (0.5 g), which was weighed into a centrifuge tube (50 mL), mixed with 20 mL of ethanol, and extracted on a horizontal laboratory shaker for 30 min at 240 revolutions per minute (RPM) according to Fathordoobady et al. (2019). After centrifugation (13,000×*g*, 5 min), the extract was removed. The sample was extracted twice using the same procedure, pooled in a volumetric flask (50 mL), and filled up to the line with ethanol. Phytocannabinoids were separated and quantified on an ultrahigh-performance liquid chromatograph (UHPLC) UltiMate 3000 system (Thermo Fisher Scientific, Pardubice Czechia), as described elsewhere (Tsagkaris et al., 2021). Briefly, it was run on the reversed-phase analytical column: Acquity UPLC BEH C18 (100 mm × 2.1 mm; 1,7 µm) (Waters, Prague, Czechia) with mobile phases of 95:5 water–methanol (v/v) and 65:30:5 isopropanol–methanol–water (v/v/v). The total run time of the method was 19 min, and the injection volume was 3 µL. The positive/negative electrospray ionization (ESI±) parameters were as follows: (i) aux gas temperature: 300°C, (ii) sheath/aux gas (N2) flow: 45/10 arbitrary units, (iii) spray voltage: 3.5 kV, and (iv) S-lens RF level: 55. Detection conditions were for full-scan MS acquisition mode: (i) resolution: 70,000 full width at half maximum (FWHM), (ii) scan range: 200–500 m/z, (iii) automatic gain control (AGC) target: 2e5, and (iv) maximum inject time (maxIT): 50 ms.

### Extraction of DNA, amplification, and sequencing

Genomic DNA was extracted from 100 mg of cleaned roots using a DNeasy Plant Mini Kit (Qiagen, Toronto, ON, Canada) and from 250 mg substrate with a DNeasy PowerSoil Pro Kit (Qiagen, Toronto, ON, Canada), according to the manufacturer’s instructions. DNA was eluted in 30 µL of elution buffer and kept at −20°C. DNA yield was measured using a NanoDropTM 2000/2000c Spectrophotometer (Thermo Fisher Scientific, Ottawa, ON, Canada) and then electrophoresed on 1% agarose gel and visualized by a GelDoc System (BioRad, Saint-Laurent, QC, Canada). Bacterial 16S rDNA and fungal ITS regions were amplified by PCR according to previously published methods (Ahmed et al., 2021a; Ahmed et al., 2021b). The PCR reaction was performed in a 25-μL reaction volume according to our previous study (Ahmed and Hijri, 2021; Ahmed et al., 2021b). We used negative PCR controls without DNA. After PCR amplification of the bacterial V3 and V4 regions of 16S rDNA and fungal ITS regions between 5.8S and 25S genes of rRNA, amplicons were electrophoresed on 1% agarose gel and visualized using GelRed on a GelDoc system (BioRad, Saint-Laurent, QC). A barcode with a unique oligonucleotide identifier (Fluidigm, Markam, ON, Canada) was added to each PCR reaction. Next, two libraries were constructed, each containing an equal amount of amplified DNA: one for bacteria and one for fungi. The pools were then purified with a 0.85 ratio of AMPure beads (Beckman Coulter, Mississauga, ON, Canada). Barcode multiplexing and amplicon sequencing on an Illumina MiSeq were performed at the Centre d’Expertise et du Service of Genome Quebec (Montreal, QC, Canada). The sequencing was performed using Illumina Reagent Kit V3 (600 cycles) with a library of 2 × 300 bp pair-end. The demultiplexing of reads was done on the instrument by the service provider.

### Sequence processing and statistical analysis

We used the DADA2 workflow (Callahan et al., 2016) operating in R4.0.2 (R core 23) to process, align, and analyze MiSeq reads. Reads were trimmed to satisfy comprehensive quality thresholds by removing primers and low-quality sequences using the filterAndTrim function (minLen = 50, maxN = 0, maxEE = c(3,3), trancQ = 2), followed by filtering with DADA2’s error simulation with the learnErrors function. We removed amplicon sequence variants (ASVs) from the bacterial and fungal databases that were taxonomically ascribed to chloroplast and mitochondria, presuming they were likely to be part of the plant genome. The fungal taxonomy dataset was cleaned of nonfungal ASVs. Taxonomy was assigned to 16S rDNA with the SILVA reference database (Quast et al., 2013) and to the ITS sequences with the UNITE database (Nilsson et al., 2018), then verified with BLASTn on NCBI. For diversity calculations, the dataset was first normalized based on the lowest number of reads for each sample with the “rarefy” function of the vegan package v 2.5-6 (Oksanen et al., 2020), before calculating the relative abundance of taxa in each family with package dplyr v2.0.0 (Wickham and Wickham, 2020). To characterize community diversity, we calculated Shannon and Simpson diversity indices as well as principal coordination analysis (PCoA) using the Bray–Curtis distance matrix of Hellinger-transformed counts with vegan package v 2.5-6. We calculated species evenness using Pielou’s index (*J* = *H*/In (*S*), where *H* is the Shannon diversity index and *S* refers the total number of species in the dataset) using vegan package v 2.5-6 on R. Dependent variables’ response to inoculant treatments for each *Cannabis* cultivar was determined by analysis of variance and Tukey’s *post-hoc* tests with the agricolae package v1.3-3 (Peşteanu and Bostan, 2020). With the function “Adonis” of the R package vegan v 2.5-6 using Hellinger-transformed and permutations 999, a permutation-based multivariate analysis of variance (PERMANOVA) (Anderson, 2001) was measured.

We visualized taxonomic abundance at the order level using metacoder v 0.3.4. (Poisot et al., 2017). According to the principles of the “core plant microbiome” (Vandenkoornhuyse et al., 2015), we identified core microbiome as taxa that are present in 100% of samples from roots or soil associated with different cultivars. We utilized the R package “RVAideMemoire” v 0.9-78 (Hervé and Hervé 2020) with the package indicspecies v 1.7.9 (De Cáceres and Jansen, 2019) using the Šidák correction for multiple comparisons. A co-occurrence network analysis was performed using the algorithm “glasso” of the SPIEC-EASI v 1.0.6 (Kurtz et al., 2015), and the findings were then visualized in Cytoscape v 3.8.0 (Shannon et al., 2003). Co-occurrences or mutual exclusions of positive or negative inverse covariance values between nodes were termed as edges. Betweenness centrality and degree emphasize central nodes as well as providing network architecture information. The ratio of the shortest path connecting all other nodes in the network, including the given node, is termed as betweenness centrality. A more than 95% ratio of betweenness centrality and degree of connectivity of network taxa may indicate community participation in multipartite co-occurrences, leading us to classify largely interconnected taxa as hub-taxa. The Venn diagrams were created with the “Calculate and make Venn diagrams” function on VIB of the University of Gent (http://bioinformatics.psb.ugent.be/webtools/Venn/). R4.0.2 (R Core Team, 2020) was used for all bioinformatics operations, including the processing of raw sequencing reads and graphical analysis.

## Results

### *Cannabis* biomass, plant height, and root colonization by *R. irregularis*

Cannabis flower biomass production varied among different cultivars. For example, when inoculated with the consortia F, containing beneficial bacteria, *Trichoderma*, and *R. irregularis*, plus microalgae, cultivars CCE and ECC produced heavier flower blossoms than cultivars THE and EEA, which produced heavier flower biomass when inoculated with K2 and K1, respectively, compared to the uninoculated control (**Supplementary Figures S1A–E**). Heavier dry flower blossoms were produced in two of the five tested cultivars when inoculated with K2. For example, 54.5 g and 59.0 g of dry biomass were produced by the cultivars THE and EEA, respectively. In contrast, three of the five tested cultivars had heavier flower biomass when they were inoculated with F, such as 101.8 g, 82.12 g, and 40 g of dry floral blossom for the cultivars CCL, ECC, and EUS, respectively (**Supplementary Figures S1A–E**). We observed the highest plant height in the ECC cultivar when inoculated with F (151.25 cm) and K1 (135 cm) treatments, whereas the EUS cultivar showed the highest plant height when inoculated with K2 (**Supplementary Figure S2A**).

The percentage of mycorrhizal colonization in cannabis plants varied significantly among cultivars. The maximum colonization rate was 19.93%, and the average number of vesicles reached six in one of the five cultivars (ECC) when inoculated with K2 compared to the control. Root mycorrhizal colonization was higher in two of the five cultivars when inoculated with K1 compared to the control plants. The cultivar had the highest average number of arbuscules (3) when inoculated with K1 and K2 compared to the control treatments (**Supplementary Figure S2C**).

### Effect of treatments on secondary metabolites production in *Cannabis* cultivars

**Figure 1:**
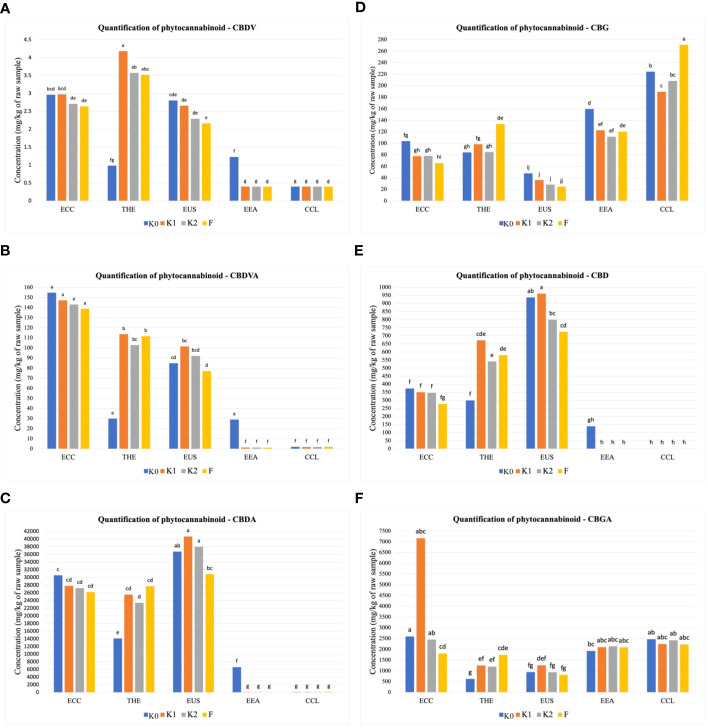
Quantitative determination of phytocannabinoids. Cumulative percent recovery of a subset of cannabinoids under different treatments in five *Cannabis* cultivars: **(A)** cannabidivarin (CBDV), **(B)** cannabidivarinic acid (CBDVA), **(C)** cannabidiolic acid (CBDA), **(D)** cannabigerol (CBG), **(E)** cannabidiol (CBD), and **(F)** cannabigerolic acid (CBGA). The bar represents the concentration in milligrams per kilogram of raw material. Values with the same letters are not significantly different by a Tukey’s range test.

**Figure 2:**
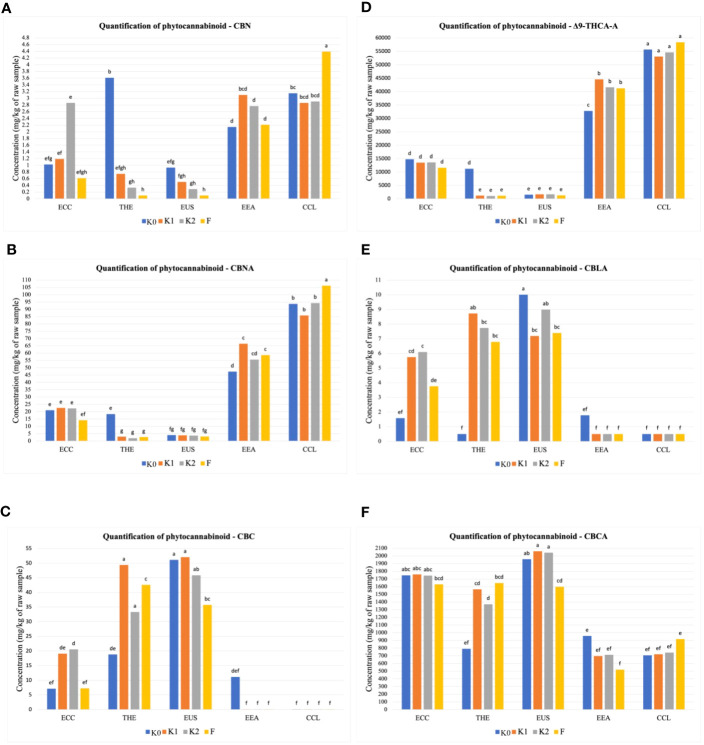
Quantitative determination of phytocannabinoids. Cumulative percent recovery of a subset of cannabinoids under different treatments in five *Cannabis* cultivars: **(A)** cannabinol (CBN), **(B)** cannabinolic acid (CBNA), **(C)** cannabichromene (CBC), **(D)** delta-9-tetrahydrocannabinol-A (△9-THCA-A), **(E)** cannabicyclolic acid (CBLA), and **(F)** cannabichromenic acid A (CBCA). The bar represents the concentration in milligrams per kilogram of raw material. Values with the same letters are not significantly different by a Tukey’s range test.

The amounts of 12 phytocannabinoid compounds quantified at the mature stage showed significant differences between treatments and cultivars. As shown in **Figure 1**, significant differences (*p* ≤ 0.01) in cannabidivarinic acid (CBDVA), cannabidivarin (CBDV), cannabigerol (CBG), cannabidiol (CBD), and cannabigerolic acid (CBGA) were observed for all cultivars when inoculated with F, K1, and K2. The cultivar THE produced higher CBDV concentration, followed by the cultivars ECC and EUS (**Figure 1A**), compared to the uninoculated control. In the cultivars ECC and EUS, CBDVA and CBDA concentrations were significantly different in all treatments (**Figures 1B, C****)**. The inoculation with Ferticann (**Figure 1D**) showed the highest increase in CBG concentrations in the CCL cultivar, while CBD was not detected with any of the treatments for the cultivar CCL (**Figure 1E**). The highest concentration of CBGA was produced in ECC when inoculated with forest microbial suspension (**Figure 1F**). However, the CCL cultivar showed a significant increase in the production of CBN (**Figure 2A**), CBNA (**Figure 2B**), and △_9_-THCA-A (**Figure 2D**) when inoculated with Ferticann, whereas CBC was not detectable for all treatments in CCL (**Figure 2C**). No significant difference was noticed among treatments with CBCA quantification (**Figure 2F**).

### Microbiome sequenced reads

We sampled roots and rhizosphere soils of five *C. sativa* cultivars to study the microbial diversity and community structure. Illumina MiSeq produced a total of 27,145,022 pair-end raw reads (8,030,182 from bacteria and 19,114,840 from fungi). The number of reads per sample ranged from 9,084 and 81,607 for bacteria and 13,499 to 194,916 for fungi. Using filtering, trimming, and quality controlling on the DADA2 pipeline resulted in 5,931 bacterial and 7,144 fungal ASVs. We also removed 11 ASVs from the bacterial dataset because they matched mitochondrial or chloroplast sequences.

### Diversity, richness, and structure in root and rhizosphere soil microbiomes across *Cannabis sativa* cultivars

**Figure 3:**
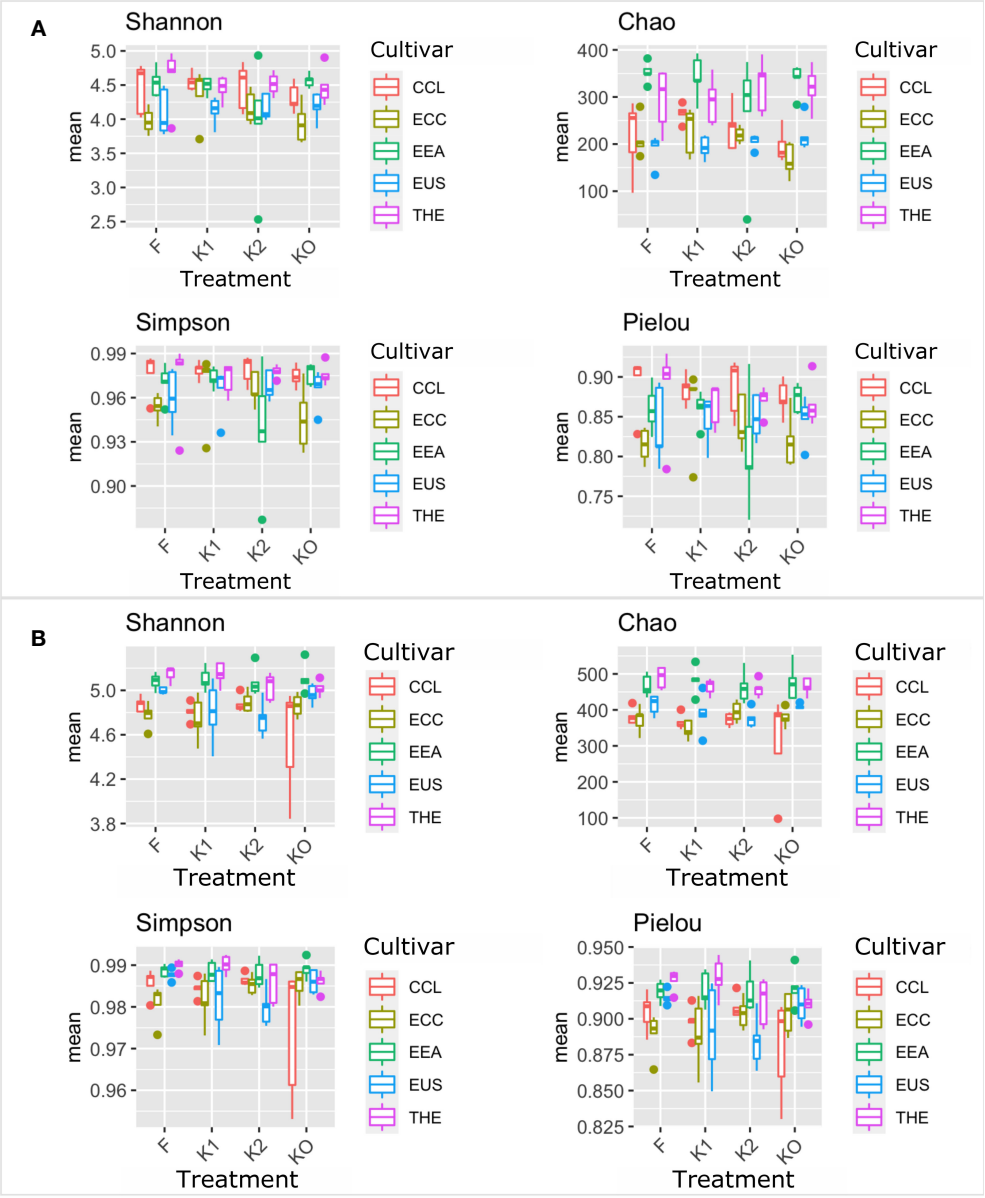
Microbial diversity and structure of the bacterial community. The analysis of alpha diversity measured by Shannon, Simpson, Chao, and Pielou of the bacterial community in roots **(A)** and rhizosphere soil **(B)**. The analysis included four *Cannabis* cultivars: CBD Therapy (THE), Euforia (EEA), Critical (CCL), CBD Sweet and Sour Widow (ECC), and CBD US (EUS) under different treatments with Ferticann (F), microbial suspension (K1), of *R. irregularis* mixed with microbial suspension (K2), and control (K0).

**Figure 4:**
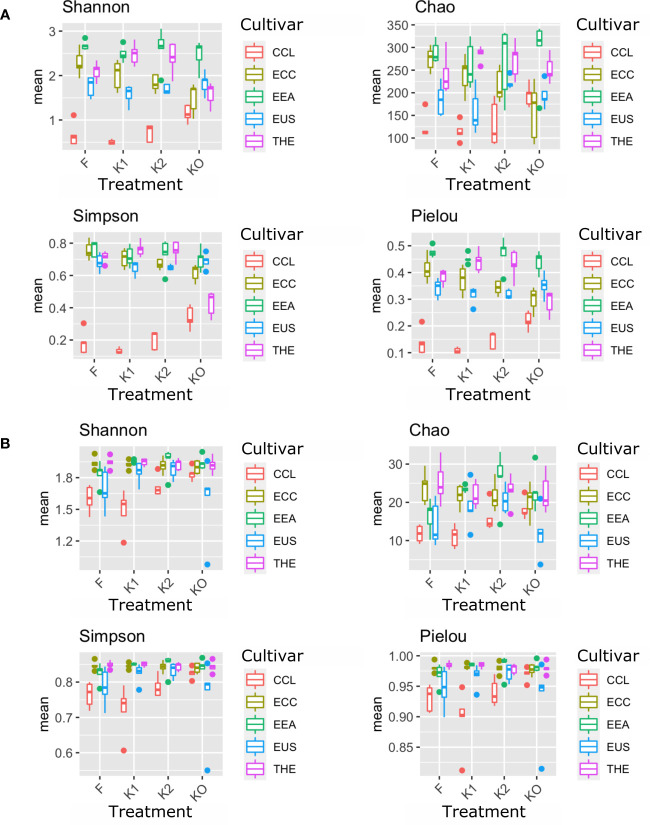
Microbial diversity and structure of the fungal community. The analysis of alpha diversity measured by Shannon, Simpson, Chao, and Pielou of the fungal community in roots **(A)** and rhizosphere soil **(B)**. The analysis included four *Cannabis* cultivars: CBD Therapy (THE), Euforia (EEA), Critical (CCL), CBD Sweet and Sour Widow (ECC), and CBD US (EUS) under different treatments with Ferticann (F), microbial suspension (K1), of *R. irregularis* mixed with microbial suspension (K2), and control (K0).

Microbial diversity indexes differed significantly among cultivars (**Figures 2**, **3**; **Table 1**). Alpha-diversity measured by Shannon, Simpson, Pielou, and Chao was significantly affected by *Cannabis* cultivars in both root and rhizosphere biotopes for bacteria (**Figure 3**) and fungi (**Figure 4**). In addition, diversity indexes Shannon (*p* = 0.0009) and Pielou (*p* = 0.044) of fungal communities in root were affected significantly by treatments (**Table 1**). Interaction between treatment and cultivar had a significant effect on alpha diversities in the rhizosphere soil microbiome. All four diversity indexes of the root fungal community were impacted by the interaction, while the interaction had a significant effect on Simpson’s evenness (*p* = 0.044) in the root bacterial community (**Table 1**).

**Table 1:** The effects of treatment and cultivar on the alpha-diversity of bacterial and fungal communities in root and rhizosphere soil.

	Indices		Root			Soil		
			Treatment	Cultivar	Treatment: cultivar	Treatment	Cultivar	Treatment: cultivar
**Bacteria**	Shannon	*F*	0.928	0.6472	1.555	0.896	17.063	2.055
		*p*-value	0.430	0.0001	0.122	0.446	3.72*E*−10	0.029
	Simpson	*F*	0.592	4.442	1.914	0.986	6.087	2.646
		*p*-value	0.621	0.002	0.044	0.403	0.0002	0.005
	Pielou	*F*	0.536	6.265	1.366	0.770	10.597	2.336
		*p*-value	0.658	0.0001	0.199	0.514	6.10*E*−07	0.012
	Chao	*F*	0.532	26.138	1.681	0.845	32.523	1.173
		*p*-value	0.661	7.12*E*−14	0.086	0.473	4.34*E*−16	0.316
**Fungi**	Shannon	*F*	2.533	139.603	6.651	1.543	19.538	3.17
		*p*-value	0.062	2.98*E*−35	4.1*E*−08	0.209	3.0*E*−11	0.0009
	Simpson	*F*	5.986	284.202	13.517	0.93	15.394	2.806
		*p*-value	0.0009	1.98*E*−46	9.96*E*−15	0.429	2.23*E*−09	0.003
	Pielou	*F*	2.805	172.462	7.757	0.781	17.035	3.087
		*p*-value	0.044	1.71*E*−38	2.4*E*−09	0.507	3.84*E*−10	0.001
	Chao	*F*	0.328	34.624	3.44	2.285	16.533	2.625
		*p*-value	0.804	9.26*E*−17	0.0004	0.085	6.53*E*−10	0.005

Beta-diversity analysis using PCoA revealed changes in microbial community structure between both cultivars and treatments (**Supplementary Figure S3**). PERMANOVA showed that the shifts in bacterial and fungal communities were highly significant (*p* < 0.002; **Table 2**). In the first two principal coordinates, the bacterial community structure showed a low percentage of variance in the root (axis 1: 7.7%, axis 2: 5.8%) (**Supplementary Figure S3A**) and rhizosphere soil (axis 1: 6.4%, axis 2: 5%) (**Supplementary Figure S3B**). Plots showed that the bacterial community in both root and rhizosphere soil did not cluster under all treatments of the five cultivars. The same was true for the fungal communities, which did not cluster differently in root (axis 1: 6.1%, axis 2: 4.1%) (**Supplementary Figure S3C**) and rhizosphere soil (axis 1: 5.7%, axis 2: 4.8%) (**Supplementary Figure S3D**). However, the cultivar EUS showed clear clustering in both bacterial (**Supplementary Figure S3A**) and fungal communities (**Supplementary Figure S3C**) in the root biotope. Significant effects of cultivar and treatments were observed both in root and rhizosphere soil in the bacterial (**Table 1**) and fungal (**Table 2**) community structure.

**Table 2:** PERMANOVA on the effects of *Cannabis* cultivar and treatments on the structure of the bacterial and fungal communities in root and rhizosphere soil biotopes.

Bacteria							
Variable	Source	*DF*	SumOfSqs	*R*^2^	*F*	*p*-value	Pr(>*F*)
Roots	Cultivar	4	3.006	0.158	4.985	0.001^***^	
	Treatment	3	0.841	0.044	1.858	0.002^**^	
	Cultivar: treatment	12	3.028	0.159	1.673	0.001^***^	
	Residual	80	12.0063	0.636			
	Total	99	18.939	1.000			
Soil	Cultivar	4	1.478	0.139	4.479	0.001^***^	
	Treatment	3	0.766	0.072	3.095	0.001^***^	
	Cultivar: treatment	12	1.722	0.162	1.739	0.001^***^	
	Residual	80	6.601	0.624			
	Total	99	10.568	1.000			
Fungi							
Variable	Source	*DF*	SumOfSqs	*R*^2^	*F*	*p*-value	Pr(>*F*)
Roots	Cultivar	4	4.863	0.302	11.773		0.001^***^
	Treatment	3	0.638	0.039	2.061		0.001^***^
	Cultivar: treatment	12	2.294	0.142	1.851		0.001^***^
	Residual	80	8.261	0.514			
	Total	99	16.057	1.000			
Soil	Cultivar	4	2.539	0.151	5.608		0.001^***^
	Treatment	3	1.982	0.118	5.838		0.001^***^
	Cultivar: treatment	12	3.088	0.185	2.273		0.001^***^
	Residual	80	9.054	0.543			
	Total	99	16.664	1.000			

*** indicates a significant difference, with *p*<0.001; ** indicates a significant difference, with *p*<0.01.

### Effect of inoculation on the microbial community assembly in root and rhizosphere soil across *Cannabis* cultivars

**Figure 5:**
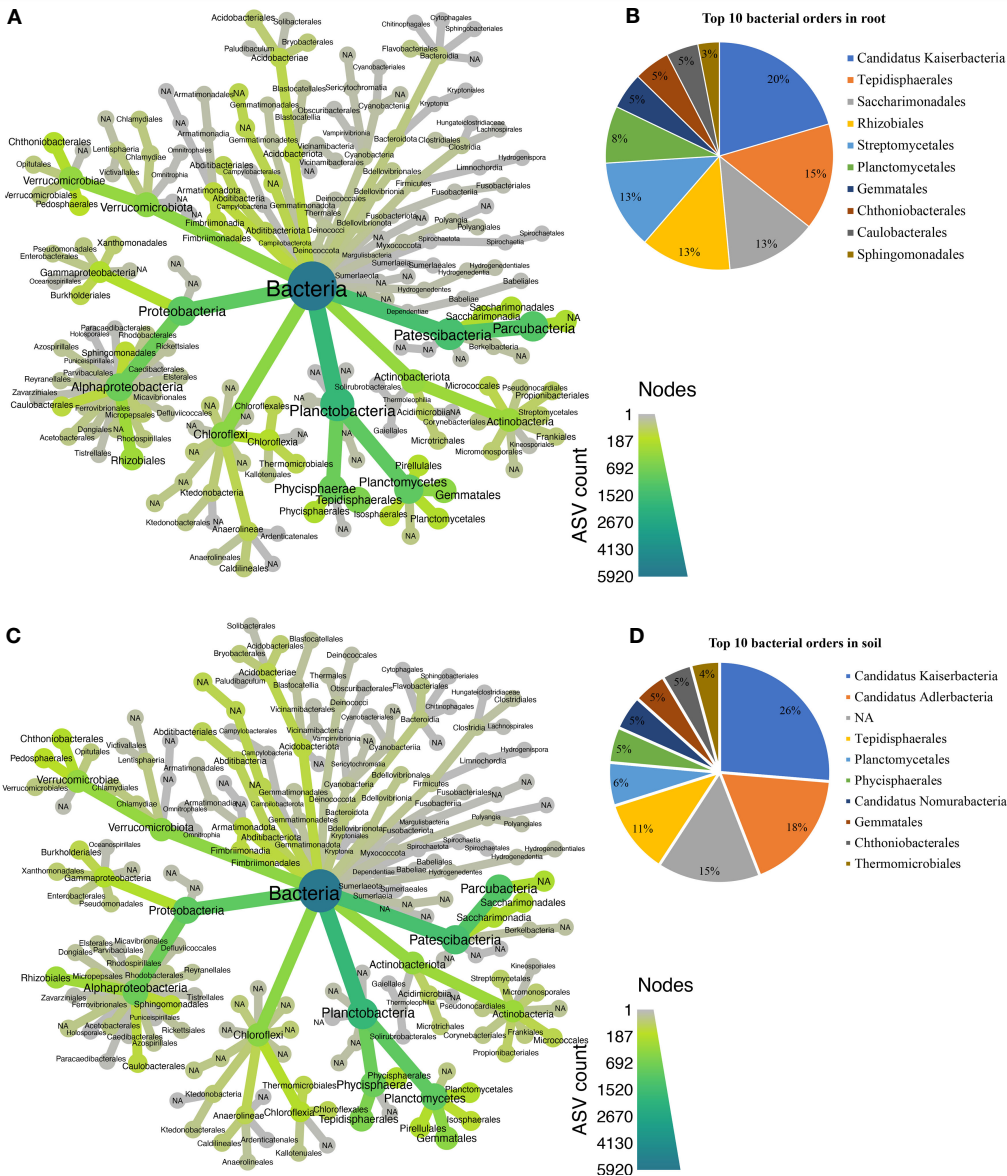
Taxonomic composition at the order level for bacterial communities. **(A)** Taxonomic representation at order level in the roots. **(B)** Relative abundance of top 10 bacterial orders in the root. **(C)** Taxonomic representation at order level in rhizosphere soil. **(D)** Relative abundance of top 10 bacterial orders in rhizosphere soil.

**Figure 6:**
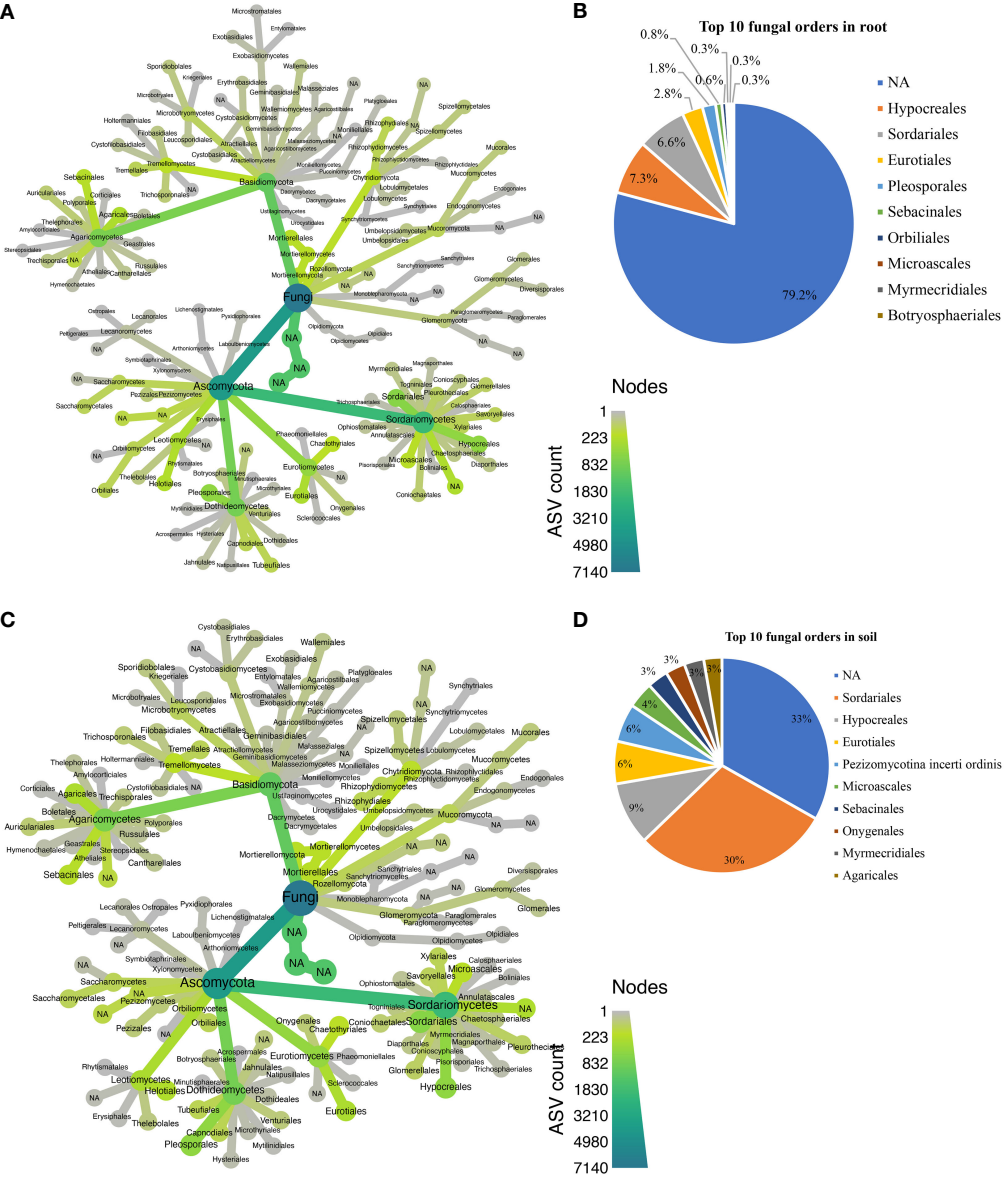
Taxonomic composition at the order level for fungal communities. **(A)** Taxonomic representation at order level in the roots; **(B)** relative abundance of top 10 fungal orders in the roots. **(C)** Taxonomic representation at order level in rhizosphere soil. **(D)** Relative abundance of top 10 fungal orders in rhizosphere soil.

Approximately 25 bacterial phyla were revealed in different biotopes (**Supplementary Table S1**), with *Patescibacteria* being the most abundant in both root (**Figure 5A**) and rhizosphere soil (**Figure 5C**). In total, 129 orders were assigned to 5,931 bacterial ASVs (**Supplementary Table S2**). Candidatus *Kaiserbacteria*, *Caulobacterales*, *Chthoniobacterales*, *Gemmatales*, *Planctomycetales*, *Rhizobiales*, *Saccharimonadales*, *Sphingomonadales*, *Streptomycetales*, and *Tepidisphaerales* were the most abundant 10 bacterial orders found in the root across all cultivars (**Figure 5B**), while the most abundant 10 orders in rhizosphere soil bacteria were Candidatus *Adlerbacteria*, Candidatus *Kaiserbacteria*, Candidatus *Nomurabacteria*, *Chthoniobacterales*, *Gemmatales*, *Phycisphaerales*, *Planctomycetales*, *Tepidisphaerales*, *Thermomicrobiales*, and not assigned to any order (NA) (**Figure 5D**). Candidatus *Kaiserbacteria* had the highest relative abundances in root and rhizosphere soil bacteria. Both biotopes shared five candidates, which include: Candidatus *Kaiserbacteria*, *Chthoniobacterales*, *Gemmatales*, *Planctomycetales*, and *Tepidisphaerales*. In the fungal dataset, we identified 11 phyla (**Supplementary Table S3**), with taxa not assigned (NA) being the most abundant in both root and rhizosphere soil across cultivars (**Figures 6A, C****)**. The fungal ASVs were classified into 125 orders, with one order remaining not assigned to any taxa. This unassigned order dominated the root and soil fungi. However, *Eurotiales*, *Hypocreales*, *Microascales*, *Mymecridiales*, *Sebacinales*, and *Sordariales* were common in both biotopes (**Figures 6B, D****)**.

### Patterns of indicator species

We found a total of 436 ASVs as indicator species across five *Cannabis* cultivars, with 195 ASVs classified as 51 unique bacterial genera and 241 ASVs identified as 77 unique fungal genera (**Supplementary Table S1**). The detailed taxonomic information of the indicator species can be found in **Supplementary Table S1**. A maximum of 49 ASVs were found in the K2 treatment in the rhizosphere soil fungal communities of the cultivar THE. *Streptomyces* sp., *Rhizobium* sp., *Bradyrhizobium* sp., *Mesorhizobium* sp., *Mesorhizobium opportunistum*, and eight different *Penicillium* spp. were identified as indicator species in the root and rhizosphere soil communities across cultivars. Three *Fusarium* spp. (*Fusarium concentricum*, *Fusarium oxysporum*, and *Fusarium solani*) were observed with the cultivars CCL, EUS, and THE. *Trichoderma reesei* was also identified in the rhizosphere soil fungi as an indicator species under the K1 treatment of the EEA cultivar (**Supplementary Table S1**).

### Eco- and core microbiome

**Figure 7:**
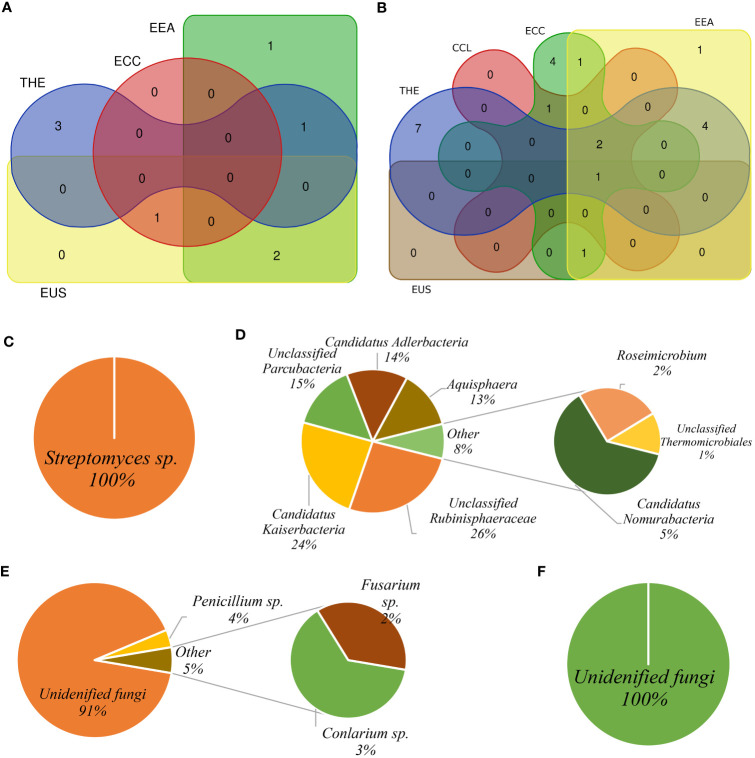
Core microbiota pattern among *Cannabis* cultivars. The Venn diagram shows eco-microbiota shared among *Cannabis* cultivars under different treatments for bacterial community **(A)** and fungal community **(B)**. Bacterial core microbiota in the root biotope **(C)** and soil biotope **(D)**. Fungal core microbiota in the root biotope **(E)** and in the rhizosphere soil biotope **(F)**.

We identified ASVs specifically associated with root and rhizosphere soil of each cultivar consisting of relatively abundant sequences that were considered eco-microbiomes in our study (**Supplementary Figures S4, S5**; **Supplementary Tables S5–S8**). In four (THE, CCL, ECC, and EUS) of the five cultivars, *Streptomycetes* sp. was the most abundant bacterial taxon in the root (**Supplementary Figures S4A–C, E**), whereas Candidatus *Kaiserbacteria* was relatively more abundant in the soil in THE, ECC, EEA, and EUS (**Supplementary Figures S5A, C–E**). Candidatus *Adlerbacteria* was identified as the most abundant soil bacterial taxon in CCL (**Supplementary Figure S5B**). This ASV was also found to be the most abundant bacterial taxon in both root and rhizosphere soil biotopes in the EEA cultivar (**Supplementary Figures S4D, S5D**). In the fungal dataset, unclassified fungi were the most abundant eco-mycobiome in root and rhizosphere soil across cultivars (**Supplementary Figures S6, S7**; **Supplementary Tables S7, S8**). Rhizosphere soil biotopes had a higher number of eco-mycobiomes than root. *Penicillium* sp. *and Fusarium* sp. were identified as root and rhizosphere soil eco-mycobiomes in cultivars THE, CCL, ECC, and EEA (**Supplementary Figures S6, S7**). We used a Venn diagram to show the distribution of eco-microbiome-related ASVs by root and rhizosphere soil and the number of ASVs shared by biotopes for each cultivar (**Supplementary Figure S8**; **Supplementary Tables S9, S10**). In the bacterial community, the Venn diagram (**Supplementary Figures S8A–E**) revealed: (i) *Asticcacaulis* sp., *Devosia* sp., and *Terrimicrobium* sp., *in* THE; (ii) *Planctomicrobium* sp. in ECC; (iii) Candidatus *Kaiserbacteria*, *Devosia* sp., and unclassified *Tepidisphaerales in* EEA; and (iv) Candidatus *Kaiserbacteria*, *Planctomicrobium* sp., and unclassified *Tepidisphaerales in* EUS. No bacterial ASV has been found common between root and rhizosphere soil in the CCL cultivar. *Devosia* sp., *Planctomicrobium* sp., and Candidatus *Kaiserbacteria* were shared between the cultivars EEA-THE, ECC-EUS, and EEA-EUS, respectively (**Figure 7A**; **Supplementary Table S9**). In the fungal dataset, unclassified fungi were more abundant in the shared eco-mycobiome list for all cultivars (**Figure 7B**; **Supplementary Table S10**). Only ASV1 (unclassified fungi) was shared by all five cultivars. ASV3 (unclassified fungi) and ASV4 (*Conlarium* sp.) were shared by four cultivars (CCL, ECC, EEA, and EUS). A maximum of four ASVs, ASV10 (*Coniochaeta* sp.), ASV12 (*F. oxysporum*), ASV15 (*F. concentricum*), and ASV24 (unclassified fungi), were found to be common between the cultivars EEA and THE (**Supplementary Table S10F**). Each cultivar has its core microbiome, which is composed of a few relatively abundant bacterial and fungal ASVs (**Figures 7C–F**; **Supplementary Table S11**). One bacterial ASV (*Streptomyces* sp.) and five fungal ASVs recognized as four taxa (unclassified fungi, *Conlarium* sp., *Penicillium citrinum*, and *F. concentricum*) were found as core microbiomes in the root (**Figures 7C, D****)**, whereas 18 ASVs identified as eight bacterial taxa (unclassified *Rubinisphaeraceae*, Candidatus *Kaiserbacteria*, unclassified *Parcubacteria*, Candidatus *Adlerbacteria*, *Aqusphaera* sp., Candidatus *Nomurabacteria*, *Roseimicrobium*, *and* unclassified *Thermomicrobiales*) and one fungal ASV (unclassified fungi) were found as core microbiomes in the rhizosphere soil (**Figures 7E, F****)**.

### Interkingdom interaction associated with five *Cannabis* cultivars

Co-occurrence network analysis of the cannabis microbiome indicated complex co-occurrence in the rhizosphere soil compared to the root. In the root, the EEA cultivar had the most co-occurrences (456 nodes and 2023 edges), while the cultivar THE had the most co-occurrences in the soil (959 nodes and 10,278 edges). We investigated the interkingdom network in the root and rhizosphere soil for each cultivar, then analyzed the meta-co-occurrence network interconnecting root and rhizosphere soil microbiomes to find hub taxa as defined by Ahmed et al. (2021b). This involved creating a module made up of a network of each hub taxon and its associated ASVs, with modular hub taxa being ASV-centered within a module. We also identified taxa that connected different modules and referred to them as connector taxa, as well as taxa that connected within modules and referred to them as network hub taxa. Detailed taxonomic information for modular hubs, connectors, and network hubs is presented in **Supplementary Table S2**. We used the terms “BASV” to refer to bacterial ASV and “FASV” to denote fungal ASV. Based on the node degree and betweenness centrality, we identified hub and network taxa in the interkingdom co-occurrence for each cultivar (**Supplementary Table S2**). Three bacterial ASVs (BASV629, BASV652, and BASV662) and two fungal ASVs (FASV373 and FASV712), a total of five ASVs, were identified as hub taxa, and 14 ASVs (six BASV and eight FASVs) were identified as connector taxa, for a total of 19 ASVs that have been designated as network hub taxa in the cultivar THE (**Supplementary Figure S9A**; **Supplementary Table S2**). The CCL cultivar had four hub taxa (one BASV and three FASVs) and six connector taxa (five BASVs and one FASV) (**Supplementary Figure S9B**; **Supplementary Table S2**). The ECC cultivar had seven hub taxa (three BASVs and four FASVs) and eight connector taxa (two BASVs and six FASVs) (**Supplementary Figure S10A**; **Supplementary Table S2**). In the EEA cultivar, four bacterial ASVs (BASV80, BASV476, BASV698, and BASV744) and one fungal ASV (FASV753) were revealed as hub taxa, and three BASVs and four FASVs were identified as connector taxa, for a total of 12 ASVs identified as network hub taxa (**Supplementary Figure S9B**; **Supplementary Table S2**). Five ASVs were found as hub taxa (three BASVs and two FASVs) with five connector taxa (three BASVs and two FASVs) in the EUS cultivar (**Supplementary Figure S11A**; **Supplementary Table S2**). We found 26 hub ASVs and 40 connector ASVs, for a total of 66 ASVs as network taxa across *Cannabis* cultivars (**Supplementary Table S2**). We also identified ASVs that were shared between cultivars, such as BASV30 (*Terrimicrobium* sp.), BASV164 (*Actinoplanes* sp.), and FASV155 (*T. reesei*), which were shared by the cultivars ECC-EUS-THE, CCL-ECC, and EUS-THE, respectively (**Supplementary Figure S11B**).

## Discussion

Previous studies reported the effectiveness of mineral nutrients (Bernstein et al., 2019; Saloner and Bernstein, 2021; Saloner and Bernstein, 2022), soil properties (Coffman and Gentner, 1975), environmental stresses (Toth et al., 2021), and biofertilizers on the biomass and phytocannabinoid properties of cannabis. This study documented that microbial suspension-based inoculants are effective in cannabis production and that cultivars responded differently to inoculation types.

### Microbial suspension-based inoculum makes the plant produce more phytocannabinoids

Secondary metabolite profile analysis by LC-MS/MS clearly showed that all treatments significantly influenced phytocannabinoid production in five *Cannabis* cultivars. Among the five cultivars tested in our trial, the cultivar THE showed the highest CBD concentration. This is consistent with previous findings that PGPR inoculum had a significant impact on the production of secondary metabolites (Pacifico et al., 2008). We highlighted the potential link between bioinoculants and cannabis cultivation, and this could serve as a baseline for further investigation.

### Microbial inoculants positively impact *Cannabis*-associated microbial community structure and recruit beneficial microbes

Determining the regulators of cannabis-associated microbial communities in the greenhouse under different treatments is a critical step in identifying beneficial microbes and establishing a microbial community structure that promotes cannabis health and productivity. Cannabis cultivation has been linked to a genotype-dependent microbiome (Winston et al., 2014; Comeau et al., 2020). For indoor-grown *Cannabis* cultivars, we showed bacterial and fungal community assemblages in the root and rhizosphere soil. Cultivars had a significant effect on the alpha-diversity of fungal communities; however, PERMANOVA revealed that treatment and cultivars had a major effect on both bacterial and fungal community structure, with findings comparable to field-grown cannabis (Ahmed et al., 2021b). Finding beneficial microbes with an impact on crop production and fitness is dependent not only on the accuracy of diversity indices but also on how they recruit and interact within the biotopes (Gould et al., 2018; Morella et al., 2020). This could be explained by the fact that multiple treatments led to greater microbial diversity as well as the ability to recruit selected microbes that could be beneficial to the host (Han et al., 2020).

The high abundance of *Patescibacteria* orders Candidatus *Kaiserbacteria* and *Tepidisphaerales* was found in our study. *Patescibacteria*, together with *Actinobacteria* and *Proteobacteria*, were recently discovered in abundance in the microbiome of chrysanthemum roots (Ma et al., 2020b). Ahmed et al. (2021a) also found that Tepidisphaerales were abundant in the soybean soil microbiome in an experiment where phytate was used as the sole source of phosphorus and a forest-microbial suspension-based amendment was applied (Ahmed et al., 2021a). Unclassified fungi were more abundant than *Ascomycota* in the fungal community, and we saw similar evidence of unclassified fungal and *Ascomycota* abundance in common bean (*Phaseolus vulgaris*) when studying the effects of amendment in a controlled environment (Barelli et al., 2020). Furthermore, *Ascomycota* was detected in high abundance in three indoor-grown cannabis chemotypes: HASH, CBD Shark, and CBD Yummy (Comeau et al., 2020).

### Inoculant-based treatment predicted cultivar-specific indicator species, cannabis core microbiome, and hub taxa

The existence and abundance of microbes can be predicted using indicator species analysis, which recognizes different bacteria and fungi and their links to cultivars (Schloter et al., 2018; Kusstatscher et al., 2019; Ahmed et al., 2021b). Our study showed microbial diversity among cultivars, with the ECC cultivar having the most indicator species. We did not find any ASV common to both the root and rhizosphere soil of the EUS cultivar. Also, ASV1 (Candidatus *Kaiserbacteria*) dominated in the root of the cultivar THE under control treatment. One possible reason for this could be the influence of root exudates on the rhizosphere soil microbiome, as soil serves as the main reservoir for rhizosphere microbes, and root exudates have been shown to shape the microbial communities in rhizosphere soil (Berg and Smalla, 2009). It is plausible that the change in community structure is due to the cultivar’s number of indicator species and the presence of beneficial microbes, including *Streptomyces* sp., *Mesorhizobium* sp., *Penicillium* sp., and *Rhizobium* sp. *Streptomyces* sp. was found as an eco-microbiome in the roots of four of the five *Cannabis* cultivar, although only bacterial core microbiome in the root and 15% of ASVs in the root eco-microbiome were *Streptomyces* sp., and it is in the Ferticann inoculum composition. *Streptomyces lincolnensi* was identified as a root indicator species in our recent studies of field-grown hemp microbiome (Ahmed et al., 2021a). *Streptomyces* sp. is being used as a biocontrol agent and a plant growth promoter (Vurukonda et al., 2018). Similarly, *P. citrinum* has been detected in several dispensary-derived cannabis samples (McKernan et al., 2016). Although *Penicillium* is known as an endophytic fungus for producing mycotoxins, *P. citrinum* has also been discovered to produce gibberellins, which are beneficial to plant growth and defense against pathogens (Khan et al., 2008). In addition to beneficial microbes, we also found *F. concentricum*, *F. oxysporium*, and *F. solani* as core microbiomes. Although *Fusarium* is known for its negative impacts on cannabis plants (Punja, 2021), its presence as a core microbiome in the bulk soil of field-grown hemp (Ahmed et al., 2021b) supports its widespread occurrence, as does studying the interactions of host-dependent pathogens to better understand the soil health of cannabis. It has been reported that *Fusarium* sp. can be detected even in *Fusarium*-suppressive soil by using the short ITS barcodes used in sequencing. The suppression is presumably due to the existence of other soil organisms such as *Actinomycetes* (Weller et al., 2002). There is no connection between the abundance of *Fusarium* in the soil and the disease caused by *Fusarium* (Vujanovic et al., 2006). *Fusarium* could be infested by beneficial bacteria, which could explain the positive connections in canola (Lay et al., 2018) and hemp (Ahmed et al., 2021b). We identified *T. reesei* as a core microbiome. Similar approaches identified *Trichoderma* sp. (*Trichoderma aerugineum*, *Trichoderma americanum*, and *Trichoderma simmonsii*) as indicator taxa in soybean (Ahmed et al., 2021a), and *T. hamatum* in the field microbiome studies in hemp (Ahmed et al., 2021b).

The interplay between a microbe and its host plant is complicated but crucial for plant performance; thus, deciphering microbial networks helps in understanding the structure and functionality of the community and how their association influences the host (Babalola et al., 2020; Ma et al., 2020a). We identified hub and connector taxa for each cultivar, totaling 66 ASVs as global hub taxa. We generated community structure and co-occurrence networks for five *Cannabis* cultivars under different microbial treatments to illustrate how microorganisms are recruited depending on genotype and treatment. Previous studies have shown the application of microbial network analysis in different plant compartments to uncover the driver microbes for plant benefits (Hamonts et al., 2018; Babalola et al., 2020; Ma et al., 2020a).

### Inoculants associated with cannabis roots were increased

Microbial diversity was diverse in K0. Since the growth substrate was a coconut coir-based medium obtained from a commercial provider, the microbial community inhabiting K0 may have provided functional diversity in our study. A recent report has found that soilless growing media have distinct community structures, stability, and functionality; for example, their organic growing medium, a mixture of white peat and coconut fiber, demonstrated unique diversity niches with temporal stability (Grunert et al., 2016). Two fungal taxa, one bacterial species, and one microalga were inoculated in two treatments (Ferticann and K2). Surprisingly, none of these microbes were found in our sequencing datasets. Although the ITS2 region is not appropriate for identifying or tracing arbuscular mycorrhizal fungi (AMF) in soils, we would expect to find sequences of *R. irregularis* in at least root biotopes in Ferticann and K2 treatments, considering the data of root colonization, which showed evidence of mycorrhizal colonization in both treatments. The K0 treatment did not show any evidence of mycorrhizal colonization, while the K1 treatment showed a very low amount of mycorrhizal colonization in some genotypes. It is likely that the root colonization observed in Ferticann and K2 resulted from *R. irregularis* inoculation, while that of the K1 treatment was due to natural propagules obtained from forest soil suspension. Furthermore, inoculum persistence was described as site-specific and reviewed by Basiru and Hijri (2022). Overall, the amount of inoculated strains decreases over time, and AMF can maintain beneficial effects on the host crop for a maximum of 4 years (Pellegrino et al., 2022). Due to competition or suppression by native communities, inoculum abundance gradually declines over time. *R. irregularis* IR27 showed 11% to 15% of the *Rhizophagus* genus in jujube roots after 13 and 18 months of inoculation. Location dependency may also affect inoculum persistence (Basiru and Hijri, 2022). In our study, AMF colonized 19.05% of the ECC cultivar’s roots (Thioye et al., 2019). To examine the fate of the AMF inoculum and establish the survival of microbial consortia for commercial reasons, we should utilize appropriate tools such as quantitative PCR, targeting specific regions (Badri et al., 2016).

## Conclusions

We found that microbial inoculants can increase the biomass, which is associated with the production of more phytocannabinoids (particularly CBDV, CBGA, and △_9_-THCA-A) while supporting a 19% greater mycorrhizal colonization in the roots of *Cannabis* cultivars. There is a core microbiome of shared bacterial and fungal taxa, with *Streptomyces* sp. always present in the roots of *Cannabis* cultivars. It appears that cannabis roots are a favorable habitat for these microorganisms, and they may be deliberately recruiting these microbiomes to support their growth, nutrition, and other biological functions. An in-depth investigation of the microbiome and transcriptomics data should enhance our understanding of the role of the cannabis-associated microbiome during plant production.

## Data availability statement

The datasets presented in this study can be found in online repositories. The names of the repository/repositories and accession number(s) can be found in the article/**Supplementary Material**.

## Author contributions

BA: conceptualization, experimental design, processing of data, and writing the manuscript. FB and JH: experimental design and data processing on phytocannabinoids. LF: conducting experiments in the greenhouse and sampling. MV and MH: concepts, design, supervision, and contribution to the manuscript writing. All authors contributed to the article and approved the submitted version.
