# Supplementary material for: Enhanced production of select phytocannabinoids in medical Cannabis cultivars using microbial consortia
Source: Front Plant Sci. 2023 Aug 31;14:1219836. doi: 10.3389/fpls.2023.1219836 (PMC10502174; doi:10.3389/fpls.2023.1219836)
Supplement: Supplementary file 1 [file DataSheet_1.docx]

**Supporting Information**

**Enhanced Phytocannabinoid Production in Medical Cannabis Cultivars using Microbial Consortia**

Bulbul Ahmed^1,2^, František Beneš^3^, Jana Hajšlová^3^, Lenka Fišarová^4^, Miroslav Vosátka^4^ and Mohamed Hijri^1,5^*

This article has the following supporting information:

**Supplementary Tables**

**Tables S1, S3-S11** are provided in Excel Format.

**Table S2. Taxonomic assignation and taxa distributions across cultivars.**

| **Cultivar** | **No. of**  **ASVs** | **Taxa type & ASVs** | **ASV ID** | **Kingdom** | **Phylum** | **Class** | **Order** | **Family** | **Genus** | **Species** |
| --- | --- | --- | --- | --- | --- | --- | --- | --- | --- | --- |
| ECC - EUS - THE | 1 | shared | BASV30 | Bacteria | Verrucomicrobiota | Verrucomicrobiae | Chthoniobacterales | Terrimicrobiaceae | *Terrimicrobium* | *Terrimicrobium sp.* |
| CCL- ECC | 1 | shared | BASV164 | Bacteria | Actinobacteriota | Actinobacteria | Micromonosporales | Micromonosporaceae | *Actinoplanes* | *Actinoplanes sp.* |
| EUS - THE | 1 | shared | FASV155 | Fungi | Ascomycota | Sordariomycetes | Hypocreales | Hypocreaceae | *Trichoderma* | *Trichoderma reesei* |
| CCL | 10 | Hub taxa (7) | BASV299 | Bacteria | Patescibacteria | Parcubacteria | Candidatus Kaiserbacteria | NA | *Candidatus Kaiserbacteria* | *Candidatus Kaiserbacteria* |
|  |  |  | FASV86 | Fungi | NA | NA | NA | NA | *Unclassified fungi* | *Unclassified fungi* |
|  |  |  | FASV204 | Fungi | Ascomycota | Sordariomycetes | Savoryellales | Savoryellaceae | *Savoryella* | *Savoryella aquatica* |
|  |  |  | FASV290 | Fungi | Basidiomycota | Tremellomycetes | Filobasidiales | Piskurozymaceae | *Solicoccozyma* | *Solicoccozyma terricola* |
|  |  |  | BASV113 | Bacteria | Patescibacteria | Parcubacteria | Candidatus Kaiserbacteria | NA | *Candidatus Kaiserbacteria* | *Candidatus Kaiserbacteria* |
|  |  |  | BASV164 | Bacteria | Actinobacteriota | Actinobacteria | Micromonosporales | Micromonosporaceae | *Actinoplanes* | *Actinoplanes sp.* |
|  |  |  | BASV48 | Bacteria | Proteobacteria | Alphaproteobacteria | Rhizobiales | Rhizobiaceae | *Rhizobium* | *Rhizobium sp.* |
|  |  | Connector (6) | FASV96 | Fungi | Ascomycota | Sordariomycetes | NA | NA | *Unclassified Sordariomycetes* | *Unclassified Sordariomycetes* |
|  |  |  | BASV180 | Bacteria | Planctobacteria | Phycisphaerae | Tepidisphaerales | WD2101 soil group | *Unclassified Tepidisphaerales* | *Unclassified Tepidisphaerales* |
|  |  |  | BASV3 | Bacteria | Patescibacteria | Parcubacteria | Candidatus Adlerbacteria | NA | *Candidatus Adlerbacteria* | *Candidatus Adlerbacteria* |
|  |  |  | BASV265 | Bacteria | Planctobacteria | Phycisphaerae | Phycisphaerales | Phycisphaeraceae | *Unclassified Phycisphaerales* | *Unclassified Phycisphaerales* |
| ECC | 15 | Hub taxa (7) | BASV127 | Bacteria | Armatimonadota | Fimbriimonadia | Fimbriimonadales | Fimbriimonadaceae | *Unclassified Fimbriimonadales* | *Unclassified Fimbriimonadales* |
|  |  |  | FASV112 | Fungi | Ascomycota | Sordariomycetes | Hypocreales | Nectriaceae | *Fusarium* | *Fusarium oxysporum* |
|  |  |  | BASV112 | Bacteria | Patescibacteria | Saccharimonadia | Saccharimonadales | LWQ8 | *Unclassified Saccharimonadales* | *Unclassified Saccharimonadales* |
|  |  |  | FASV324 | Fungi | Basidiomycota | Agaricomycetes | NA | NA | *Unclassified Agaricomycetes* | *Unclassified Agaricomycetes* |
|  |  |  | BASV15 | Bacteria | Planctobacteria | Planctomycetes | Planctomycetales | Rubinisphaeraceae | *Unclassified Rubinisphaeraceae* | *Unclassified Rubinisphaeraceae* |
|  |  |  | FASV11 | Fungi | Ascomycota | Sordariomycetes | Hypocreales | Nectriaceae | *Fusarium* | *Fusarium oxysporum* |
|  |  |  | FASV67 | Fungi | Ascomycota | Sordariomycetes | Sordariales | Sordariales_fam_Incertae_sedis | *Conlarium* | *Conlarium sp.* |
|  |  | Connector (8) | BASV44 | Bacteria | Patescibacteria | Saccharimonadia | Saccharimonadales | Saccharimonadaceae | *Unclassified Saccharimonadales* | *Unclassified Saccharimonadales* |
|  |  |  | FASV83 | Fungi | Ascomycota | Pezizomycotina_cls_Incertae_sedis | Pezizomycotina_ord_Incertae_sedis | Pezizomycotina_fam_Incertae_sedis | *Ciliophora* | *Ciliophora sp.* |
|  |  |  | BASV201 | Bacteria | Proteobacteria | Alphaproteobacteria | Rhizobiales | Rhizobiaceae | *Shinella* | *Shinella fusca* |
|  |  |  | FASV82 | Fungi | Ascomycota | Dothideomycetes | Botryosphaeriales | Botryosphaeriaceae | *Neodeightonia* | *Neodeightonia phoenicum* |
|  |  |  | FASV89 | Fungi | Ascomycota | Sordariomycetes | Sordariales | Chaetomiaceae | *Zopfiella* | *Zopfiella marina* |
|  |  |  | FASV329 | Fungi | Ascomycota | Sordariomycetes | Sordariales | Chaetomiaceae | *Trichocladium* | *Trichocladium pyriforme* |
|  |  |  | FASV54 | Fungi | Ascomycota | Eurotiomycetes | Eurotiales | Aspergillaceae | *Penicillium* | *Penicillium sp.* |
|  |  |  | FASV51 | Fungi | Ascomycota | Eurotiomycetes | Eurotiales | Aspergillaceae | *Penicillium* | *Penicillium pimiteouiense* |
| EEA | 12 | Hub taxa (5) | BASV744 | Bacteria | Gemmatimonadota | Gemmatimonadetes | Gemmatimonadales | Gemmatimonadaceae | *Unclassified Gemmatimonadales* | *Unclassified Gemmatimonadales* |
|  |  |  | BASV80 | Bacteria | Planctobacteria | Planctomycetes | Gemmatales | Gemmataceae | *Fimbriiglobus* | *Fimbriiglobus sp.* |
|  |  |  | BASV698 | Bacteria | Planctobacteria | Planctomycetes | Gemmatales | Gemmataceae | *Unclassified Gemmatales* | *Unclassified Gemmatales* |
|  |  |  | BASV476 | Bacteria | Planctobacteria | Phycisphaerae | Tepidisphaerales | Tepidisphaeraceae | *Unclassified Tepidisphaerales* | *Unclassified Tepidisphaerales* |
|  |  |  | FASV753 | Fungi | Basidiomycota | Agaricomycetes | Sebacinales | NA | *Unclassified Sebacinales* | *Unclassified Sebacinales* |
|  |  | Connector (7) | BASV452 | Bacteria | Proteobacteria | Alphaproteobacteria | Rhizobiales | Hyphomicrobiaceae | *Hyphomicrobium* | *Hyphomicrobium sp.* |
|  |  |  | FASV175 | Fungi | Basidiomycota | Agaricomycetes | Sebacinales | NA | *Unclassified Sebacinales* | *Unclassified Sebacinales* |
|  |  |  | BASV279 | Bacteria | Proteobacteria | Alphaproteobacteria | Rhizobiales | Xanthobacteraceae | *Pseudolabrys* | *Pseudolabrys sp.* |
|  |  |  | BASV37 | Bacteria | Patescibacteria | Parcubacteria | Candidatus Kaiserbacteria | NA | *Candidatus Kaiserbacteria* | *Candidatus Kaiserbacteria* |
|  |  |  | FASV36 | Fungi | Ascomycota | Sordariomycetes | Sordariales | Chaetomiaceae | *Zopfiella* | *Zopfiella marina* |
|  |  |  | FASV85 | Fungi | Ascomycota | Dothideomycetes | Botryosphaeriales | Botryosphaeriaceae | *Lasiodiplodia* | *Lasiodiplodia sp.* |
|  |  |  | FASV911 | Fungi | Basidiomycota | Agaricomycetes | Agaricomycetes_ord_Incertae_sedis | Agaricomycetes_fam_Incertae_sedis | *Xenasmatella* | *Xenasmatella sp.* |
| EUS | 10 | Hub taxa (5) | BASV410 | Bacteria | Actinobacteriota | Actinobacteria | Micrococcales | Microbacteriaceae | *Leifsonia* | *Leifsonia sp.* |
|  |  |  | BASV98 | Bacteria | Proteobacteria | Alphaproteobacteria | Caulobacterales | Caulobacteraceae | *Asticcacaulis* | *Asticcacaulis sp.* |
|  |  |  | FASV132 | Fungi | Ascomycota | Sordariomycetes | Glomerellales | Plectosphaerellaceae | *Plectosphaerella* | *Plectosphaerella sp.* |
|  |  |  | FASV155 | Fungi | Ascomycota | Sordariomycetes | Hypocreales | Hypocreaceae | *Trichoderma* | *Trichoderma reesei* |
|  |  |  | BASV30 | Bacteria | Verrucomicrobiota | Verrucomicrobiae | Chthoniobacterales | Terrimicrobiaceae | *Terrimicrobium* | *Terrimicrobium sp.* |
|  |  | Connector (5) | BASV759 | Bacteria | Chloroflexi | Chloroflexia | Kallotenuales | AKIW781 | *Unclassified Kallotenuales* | *Unclassified Kallotenuales* |
|  |  |  | FASV53 | Fungi | Ascomycota | Sordariomycetes | Myrmecridiales | Myrmecridiales_fam_Incertae_sedis | *Atractospora* | *Atractospora sp.* |
|  |  |  | FASV62 | Fungi | Ascomycota | Sordariomycetes | Sordariales | Chaetomiaceae | *Humicola* | *Humicola sp.* |
|  |  |  | BASV208 | Bacteria | Proteobacteria | Alphaproteobacteria | Sphingomonadales | Sphingomonadaceae | *Unclassified Sphingomonadales* | *Unclassified Sphingomonadales* |
|  |  |  | FASV516 | Fungi | Ascomycota | Eurotiomycetes | Eurotiales | Aspergillaceae | *Penicillium* | *Penicillium sp.* |
| THE | 19 | Hub taxa (5) | BASV662 | Bacteria | Planctobacteria | Planctomycetes | Gemmatales | Gemmataceae | *Unclassified Gemmatales* | *Unclassified Gemmatales* |
|  |  |  | FASV373 | Fungi | Basidiomycota | Cystobasidiomycetes | Erythrobasidiales | Erythrobasidiales_fam_Incertae_sedis | *Sakaguchia* | *Sakaguchia sp.* |
|  |  |  | FASV712 | Fungi | Ascomycota | Leotiomycetes | Helotiales | Helotiaceae | *Meliniomyces* | *Meliniomyces sp.* |
|  |  |  | BASV629 | Bacteria | Patescibacteria | Parcubacteria | Candidatus Kaiserbacteria | NA | *Candidatus Kaiserbacteria* | *Candidatus Kaiserbacteria* |
|  |  |  | BASV652 | Bacteria | Chloroflexi | Anaerolineae | Caldilineales | Caldilineaceae | *Unclassified Caldilineales* | *Unclassified Caldilineales* |
|  |  | Connector (14) | FASV220 | Fungi | Ascomycota | Dothideomycetes | Capnodiales | Mycosphaerellaceae | *Acrodontium* | *Acrodontium hydnicola* |
|  |  |  | FASV832 | Fungi | NA | NA | NA | NA | *Unclassified fungi* | *Unclassified fungi* |
|  |  |  | FASV179 | Fungi | Ascomycota | Sordariomycetes | Sordariales | Chaetomiaceae | *Unclassified Sordariomycetes* | *Unclassified Sordariomycetes* |
|  |  |  | BASV280 | Bacteria | Patescibacteria | Parcubacteria | NA | NA | *Unclassified Parcubacteria* | *Unclassified Parcubacteria* |
|  |  |  | FASV917 | Fungi | Ascomycota | Eurotiomycetes | Chaetothyriales | Herpotrichiellaceae | *Exophiala* | *Exophiala xenobiotica* |
|  |  |  | BASV458 | Bacteria | Patescibacteria | Parcubacteria | Candidatus Kaiserbacteria | NA | *Candidatus Kaiserbacteria* | *Candidatus Kaiserbacteria* |
|  |  |  | FASV527 | Fungi | Ascomycota | Sordariomycetes | Sordariales | Chaetomiaceae | *Unclassified Sordariomycetes* | *Unclassified Sordariomycetes* |
|  |  |  | BASV1048 | Bacteria | Proteobacteria | Alphaproteobacteria | Micropepsales | Micropepsaceae | *Unclassified Micropepsales* | *Unclassified Micropepsales* |
|  |  |  | BASV166 | Bacteria | Proteobacteria | Alphaproteobacteria | Rhizobiales | Xanthobacteraceae | *Afipia* | *Afipia felis* |
|  |  |  | FASV485 | Fungi | NA | NA | NA | NA | *Unclassified fungi* | *Unclassified fungi* |
|  |  |  | BASV129 | Fungi | Ascomycota | Eurotiomycetes | Eurotiales | Aspergillaceae | *Penicillium* | *Penicillium riverlandense* |
|  |  |  | BASV604 | Bacteria | Chloroflexi | Anaerolineae | Caldilineales | Caldilineaceae | *Unclassified Caldilineales* | *Unclassified Caldilineales* |
|  |  |  | FASV139 | Fungi | Basidiomycota | Microbotryomycetes | Sporidiobolales | Sporidiobolaceae | *Rhodotorula* | *Rhodotorula toruloides* |
|  |  |  | FASV243 | Fungi | Ascomycota | Dothideomycetes | Pleosporales | NA | *Unclassified Pleosporales* | *Unclassified Pleosporales* |

**Supplementary Figures:**

**Supplementary Figure 1. Cannabis flower biomass.** Fresh weight (in green) and dry weight (in grey) of the flower blossom in five cannabis cultivars: (A) THE; (B) CCL; (C) ECC; (D) EEA and (E) EUS. *Cannabis* plants were inoculated with Ferticann (F) containing beneficial bacteria, *Trichoderma,* and *R. irregularis* plus microalgae; forest microbial suspension plus *R. irregularis* (K2) and forest microbial suspension (K1). With each treatment group, means with the same letter are not significantly different (P> 0.05) by a Tukey's range test. THE = CBD Therapy; CCL = Critical; ECC = CBD Sweet and Sour Widow; EEA = Euforia and EUS = CBD US.

**Supplementary Figure 2. Cannabis biomass and mycorrhizal colonization of roots, in response to microbial inoculation.** (A) Cannabis height (cm) after 90 days growth, with no inoculation (KO) or inoculation with Ferticann (F) containing beneficial bacteria, *Trichoderma,* and *R. irregularis* plus microalgae; forest microbial suspension plus *R. irregularis* (K2) and forest microbial suspension (K1). CCL = Critical, ECC **=** CBD Sweet and Sour Widow, EEA = Euforia, EUS = CBD US and THE = CBD Therapy. (B) Mycorrhizal colonization of cannabis roots showing typical AMF vesicles and intraradical hyphae stained with Cotton blue. (C) Estimation of mycorrhizal colonization in the root system. Presence of mycorrhizal fungi, average number of arbuscules and vesicles, and % of root colonization are presented in different colours.

**Supplementary Figure 3.** Principle coordinates analysis (PCoA) of bacterial diversity in root (A) and rhizosphere soil (B); fungal diversity in root (C) and rhizosphere soil (D). Community composition assignments of cannabis cultivars and treatments are done with PCoA. The percentage of the total variance explained by each axis is shown in parentheses. Each color represents cannabis cultivar, CCL = Critical, ECC **=** CBD Sweet and Sour Widow, EEA = Euforia, EUS = CBD US and THE = CBD Therapy. Each shape represents treatments, F = Ferticann, K1 = microbial suspension, K2 = *R. irregularis* mixed with microbial suspension and KO = control.

**Supplementary Figure 4. Bacterial eco-microbiota in root.** Relative abundance of the bacterial eco-microbiota at the species level in root of cannabis cultivars. CCL = Critical, ECC **=** CBD Sweet and Sour Widow, EEA = Euforia, EUS = CBD US and THE = CBD Therapy.

**Supplementary Figure 5. Bacterial eco-microbiota in rhizosphere soil.** Relative abundance of the bacterial eco-microbiota composition at the species level in rhizosphere soil of cannabis cultivars. CCL = Critical, ECC **=** CBD Sweet and Sour Widow, EEA = Euforia, EUS = CBD US and THE = CBD Therapy.

**Supplementary Figure 6. Fungal eco-mycobiota in root.** Pie chart showing fungal, eco-mycobiota composition (relative abundance) at species level in root in different cannabis cultivars: THE (A), CCL (B), ECC (C), EEA (D) and EUS (E). CCL = Critical, ECC **=** CBD Sweet and Sour Widow, EEA = Euforia, EUS = CBD US and THE = CBD Therapy.

**Supplementary Figure 7. Fungal eco-mycobiota in rhizosphere soil.** Pie chart showing fungal, eco-mycobiota composition (relative abundance) at species level in rhizosphere soil in different cannabis cultivars: THE (A), CCL (B), ECC (C), EEA (D) and EUS (E). CCL = Critical, ECC **=** CBD Sweet and Sour Widow, EEA = Euforia, EUS = CBD US and THE = CBD Therapy.

**Supplementary Figure 8. Amplicon Sequence Variant (ASV) shared by biotopes in each cannabis cultivar.** The Venn diagram shows the number of ASVs found unique or shared in root: (A) THE = CBD Therapy; (B) CCL = Critical; (C) ECC **=** CBD Sweet and Sour Widow; (D) EEA = Euforia and (E) EUS = CBD US. The number of ASVs found unique or shared in rhizosphere soil in (A) THE = CBD Therapy; (B) CCL = Critical; (C) ECC **=** CBD Sweet and Sour Widow; (D) EEA = Euforia and (E) EUS = CBD US.

**Supplementary Figure 9. Network cooccurrence in THE and CCL cultivar.** (A) Interkingdom network patterns in CBD Therapy (THE). Three bacterial and two fungal ASVs were identified as hub taxa, 14 connector ASVs, total 19 ASVs were identified as network hub in THE cultivar. (B) network cooccurrence patterns in Critical (CCL). Ten hub taxa included four hub taxa and six connector taxa. Bacterial (circular) and fungal (rhombus) ASVs are shown by the different node shape. The relative abundance of the corresponding ASVs determines how nodes are coloured. The ribbon depicts the relative complexity of the inter-kingdom network. Betweenness centrality and node degree of ASVs and their inter-connection were considered in identifying hub taxa.

**Supplementary Figure 10. Network cooccurrence in ECC and EEA cultivar.** (A) Interkingdom network patterns in CBD Sweet and Sour Widow (ECC). Three bacterial and four fungal ASVs were identified as hub taxa, and eight connector ASVs were identified as network hub in ECC cultivar. (B) network cooccurrence patterns in Euforia (EEA). Twelve ASVs were identified as network hub included four bacterial and one fungal ASVs as hub taxa and three bacterial and four fungal ASVs as connector taxa. Bacterial (circular) and fungal (rhombus) ASVs are shown by the different node shape. The relative abundance of the corresponding ASVs determines how nodes are coloured. The ribbon depicts the relative complexity of the inter-kingdom network. Betweenness centrality and node degree of ASVs and their inter-connection were considered in identifying hub taxa.

**Supplementary Figure 11. Network cooccurrence in EUS cultivar and network taxa shared across cultivars.** (A) Interkingdom network patterns in CBD US (EUS). Five ASVs were found as hub taxa (three bacterial ASVs and two fungal ASVs) with five connector taxa in the EUS cultivar. Bacterial (circular) and fungal (rhombus) ASVs are shown by the different node shape. The relative abundance of the corresponding ASVs determines how nodes are coloured. The ribbon depicts the relative complexity of the inter-kingdom network. Betweenness centrality and node degree of ASVs and their inter-connection were considered in identifying hub taxa. (B) ASVs shared between cultivars in the network cooccurrence pattern. 26 hub ASVs and 40 connector ASVs, total 66 ASVs were identified as network taxa across *Cannabis* cultivars (details in Table 4).

**Fig. S1**

**Fig. S2.**

**Fig. S3.**

**Fig. S4.**

**Fig. S5.**

**Fig. S6.**

**Fig. S7.**

**Fig. S8.**

**Fig. S9.**

**Fig. S10.**

**Fig. S11.**
